# ﻿Morphological characteristics and phylogenetic analysis reveal *Helvellosebacinafilicata* sp. nov. and *Sebacinaaciculicola* sp. nov. in Sebacinales from southwest China

**DOI:** 10.3897/mycokeys.118.152160

**Published:** 2025-06-03

**Authors:** Jia-Hui Dong, Xin Zhang, Qian-Xin Guan, Fang Wu

**Affiliations:** 1 State Key Laboratory of Efficient Production of Forest Resources, School of Ecology and Nature Conservation, Beijing Forestry University, Beijing 100083, China Beijing Forestry University Beijing China

**Keywords:** Basidiomycota, fungal diversity, new taxa, Sebacinaceae, taxonomy

## Abstract

Morphological characteristics and phylogenetic analyses based on ITS and nLSU sequences reveal two new species of Sebacinaceae from southwest China, viz. *Helvellosebacinafilicata***sp. nov.** and *Sebacinaaciculicola***sp. nov.** The two species have similar micromorphology characterized by clampless hyphae, hyphidia with simple septa, longitudinally septate, 4-celled, ovoid to subglobose basidia, and broadly ellipsoid to ovoid basidiospores. However, *H.filicata* is characterized by cartilaginous to gelatinous, white to cream, sometimes semi-transparent basidiomata; basidiospores measuring 8.6–9.9 × 5.8–7.6 μm; and growth on dead ferns. *S.aciculicola* is characterized by coriaceous, white-to-cream basidiomata; basidiospores measuring 10.2–12.9 × 6.3–8.7 μm; and growth on needles and bark of *Pinus*. The two new species are described and illustrated in this study.

## ﻿Introduction

The order Sebacinales is a recently described group separated from Auriculariales based on molecular analysis (Weiss et al. 2004). It includes the two well-supported families Sebacinaceae and Serendipitaceae (Weiß et al. 2016). The family Sebacinaceae was proposed to include *Sebacina* s. str., *Tremelloscypha* D.A. Reid, and *Tremellodendron* G.F. Atk. *Efibulobasidium* K. Wells was also tentatively assigned to the family (Wells and Oberwinkler 1982). However, *Tremellodendron* was merged into *Sebacina* Tul. & C. Tul., the extant genera *Chaetospermum* Sacc. and *Craterocolla* Bref. were moved into the family, and three genera, viz., *Globulisebacina* Oberw. et al., *Helvellosebacina* Oberw. et al., and *Paulisebacina* Oberw. et al., were newly proposed to accommodate some species formerly placed in *Sebacina* by phylogenetic analysis of the family (Oberwinkler et al. 2014). Malysheva et al. (2019) argued that *Ditangium* P. Karst. should be restored as a correct genus and that *Craterocolla* should be treated as its younger synonym by showing that the type species of the two genera were conspecific. Nowadays, eight genera, viz. *Chaetospermum*, *Ditangium*, *Efibulobasidium*, *Globulisebacina*, *Helvellosebacina*, *Paulisebacina*, *Sebacina*, and *Tremelloscypha*, are recognized in the family Sebacinaceae (He et al. 2024, http://www.indexfungorum.org).

The family Sebacinaceae, typified by *Sebacina*, is characterized by coriaceous, waxy, cartilaginous, gelatinous, subglobose, pustulate, cerebriform, helvelloid basidiomata, clampless hyphae, abundant hyphidia (dikaryophyses), longitudinally or obliquely septate, obovate to subglobose basidia, and allantoid, cylindrical, ellipsoid, or ovoid basidiospores (Tulasne and Tulasne 1871; Wells and Oberwinkler 1982; Oberwinkler et al. 2014; Malysheva et al. 2019). *Sebacina* was established using *S.incrustans* (Pers.) Tul. & C. Tul. with waxy and resupinate basidiomata as the type species (Tulasne and Tulasne 1871). It grows in clusters on the ground and on dead branches and leaves in broadleaf forests or mixed broadleaf-coniferous forests, forming ECM associations with diverse plants. It is common in Europe, North America, and parts of Asia (Tedersoo et al. 2014; Garnica et al. 2016). *Helvellosebacina* was established by Oberwinkler et al. (2014), with *H.concrescens* (Schwein.) Oberw. as the type species, and this species is characterized by gelatinous and granular basidiomata. Members of this genus typically occur either solitarily or in groups in deciduous forests, where they form associations with diverse plant hosts, including ferns, *Carpinus*, *Carpinusorientalis* (Betulaceae), and *Corylusavellana* (Betulaceae) (Sesli 2021). The genus is widely distributed in Asia, Europe, North America, and South America.

*Chaetospermum* is characterized by pycnidial conidiomata, holoblastic conidiogenesis, and hyaline, cylindrical, non-septate, slimy conidia with tubular appendages at both ends (Tangthirasunun et al. 2014). *Efibulobasidium* morphologically is similar to *Chaetospermum*, but it produces gelatinous basidiomata without conidial stages, and the two genera formed separate monophyletic clades in the phylogenies (Rungjindamai et al. 2008; Kirschner et al. 2009). *Ditangium* has cerebriform to pustulate pinkish basidiomata, occasionally accompanied by conidial formation (Malysheva et al. 2019). *Globulisebacina* has pustulate or cup-shaped basidiomata, and *Tremelloscypha* produces cup-shaped or flat-expanded basidiomata (Oberwinkler et al. 2014). *Paulisebacina* is characterized by minute, hardly visible basidiomata, small basidia, and allantoid basidiospores (Kirschner et al. 2002).

In this study, specimens morphologically corresponding to the concept of the family Sebacinaceae were collected from southwest China. A phylogenetic analysis based on ITS and nLSU sequences was conducted to confirm their affinity. Phylogenetically, these samples formed two distinct lineages within *Helvellosebacina* and *Sebacina*, respectively, and they are different from their morphologically similar and phylogenetically related species. Therefore, the two new species, *Helvellosebacinafilicata* and *Sebacinaaciculicola* in the Sebacinaceae, are described based on morphological studies and phylogenetic analyses.

## ﻿Materials and methods

### ﻿Morphological studies

The studied specimens are deposited in the Fungarium of Beijing Forestry University (BJFC). Field notes and voucher herbarium specimens served as the foundation for macro-morphological descriptions. 5% KOH and 2% Phloxine B (C20H2Br4Cl4Na2O5) were used to make slides from voucher tissues for microscopic measurements and drawings. Special color terms follow Petersen (1996). The following abbreviations were used: KOH = 5% potassium hydroxide; L = mean basidiospore length (arithmetic average of basidiospores); W = mean basidiospore width (arithmetic average of basidiospores); Q = spore L/W ratios of the specimens studied; and n (a/b) = number of basidiospores (a) measured from the given number of specimens (b). The values supplied in parentheses correspond to mean values with 5% of the measurements removed from both ends of the range.

### ﻿DNA extraction and sequencing

DNA was extracted from dried specimens using a rapid plant genome extraction kit (Aidlab Biotechnologies Co., Ltd., Beijing, China) with some modifications (Wu et al. 2015, 2022). The nuclear ribosomal internal transcribed spacer (ITS) region and the large subunit of the nuclear ribosomal RNA operon (nLSU) were amplified with primer pairs ITS 4 and ITS 5 (White et al. 1990) and LROR and LR7 (Vilgalys and Hester 1990), respectively. The PCR (polymerase chain reaction) procedure for ITS was initial denaturation at 95 °C for 3 min, followed by 35 cycles at 94 °C for 40 s, 54 °C for 45 s, and 72 °C for 1 min, and a final extension of 72 °C for 10 min. The PCR procedure for nLSU was initial denaturation at 94 °C for 1 min, followed by 35 cycles at 94 °C for 1 min, 50 °C for 1 min, and 72 °C for 1 min, and a final extension of 72 °C for 10 min. The PCR products were purified and sequenced at the Beijing Genomics Institute, China, with the same primers used in the PCR reactions. The newly generated sequences were detailed in Table 1 and were deposited in GenBank (Sayers et al. 2025).

**Table 1.** Taxa information and GenBank accession numbers of sequences used in this study.

Species name	Samples/Voucher	Country	GenBank accessions		Reference
			ITS	nLSU	
*Chaetospermumartocarpi*	MFLUCC12-0536	France	KF516968	KF516974	Tangthirasunun et al. (2014)
*Chaetospermumcamelliae*	CL-1	China	JQ794486	JQ794488	Tan et al. (2014)
*Chaetospermumcamelliae*	MFLUCC12-0318	France	KF516964	KF516970	Tangthirasunun et al. (2014)
*Chaetospermumcamelliae*	MFLUCC12-0433	France	KF516965	KF516971	Tangthirasunun et al. (2014)
*Chaetospermumchaetosporum*	Soil_F52	India	PQ552916	–	Nyayiru Kannaian et al. (2024)
*Chaetospermumchaetosporum*	Bin_112	India	PQ552916	–	Nyayiru Kannaian et al. (2024)
*Chaetospermumgossypinum*	13RBGC6	Ireland	KF307622	–	Murphy et al (2014)
*Chaetospermum* sp.	TUB 020201	Germany	KF061263	KF061263	Oberwinkler et al. (2014)
*Ditangiumaltaicum*	LE 231836*	Russia	MH836338	–	Malysheva et al. (2019)
*Ditangiumcerasi*	Karsten 3508	Finland	MH836341	MH836341	Malysheva et al. (2019)
*Ditangiumcerasi*	TUB 020203	Germany	KF061265	KF061265	Oberwinkler et al. (2014)
*Ditangiumcerasi*	TUB 020202	Germany	KF061264	KF061264	Oberwinkler et al. (2014)
*Ditangiumincarnatum*	LE 303419	Russia	MH836337	–	Malysheva et al. (2019)
*Ditangiumincarnatum*	LE 206311	Russia	MH836336	–	Malysheva et al. (2019)
*Efibulobasidiumalbescens*	RJB 12952	Canada	AF384860	AF384860	–
*Globulisebacinarolleyi*	RJB794	Canada	AY509550	AY509550	Wells et al. (2004)
*Helvellosebacinaconcrescens*	TUB 019706	Germany	JQ665516	JQ665516	Riess et al. (2013)
*Helvellosebacinaconcrescens*	27.1	Russia	MG844990	MG844990	Dudka et al. (2023)
*Helvellosebacinaconcrescens*	27.2	Russia	MG844991	MG844991	Dudka et al. (2023)
*Helvellosebacinaconcrescens*	27.3	Russia	MG844992	MG844992	Dudka et al. (2023)
*Helvellosebacinaconcrescens*	132289	Ireland	KF307625	–	Murphy et al (2014)
***Helvellosebacinafilicata***	Dai 20449*	**China**	**PQ877259**	**PQ877258**	**this study**
*Helvellosebacinafilicata*	SeqID44	China	OR482700	OR482700	–
*Helvellosebacinagranulata*	KATO F. 4080*	Turkey	MT302587	MT302588	Sesli (2021)
*Helvellosebacinahelvelloides*	TUB 019707	Germany	JQ665515	JQ665515	Riess et al. (2013)
*Helvellosebacinahelvelloides*	TUB 019983	Germany	KF000415	KF000415	Riess et al. (2013)
*Helvellosebacinahelvelloides*	TUB 019984	Germany	KF000416	KF000416	Riess et al. (2013)
*Helvellosebacinahelvelloides*	TUB 020031	Germany	KF000459	KF000459	Riess et al. (2013)
*Helvellosebacinahelvelloides*	TUB 020032	Germany	KF000460	KF000460	Riess et al. (2013)
*Helvellosebacinahelvelloides*	TUB 020033	Germany	KF000461	KF000461	Riess et al. (2013)
*Helvellosebacinahelvelloides*	TUB:019681	Germany	KJ546097	KJ546097	Oberwinkler et al. (2014)
*Helvellosebacina* sp.	TUB 020021	Germany	KF000449	KF000449	Oberwinkler et al. (2014)
*Helvellosebacina* sp.	TUB 020028	Germany	KF000456	KF000456	Oberwinkler et al. (2014)
*Paulisebacinaallantoidea*	RoKi 179*	Germany	KF061266	AF291367	Oberwinkler et al. (2014)
***Sebacinaaciculicola***	Dai 25793*	**China**	**PQ877260**	–	**this study**
*Sebacinaaciculicola*	SeqID43	China	OR482699	OR482699	–
*Sebacinacandida*	TUB 020330	USA	KF061277	KF061277	Oberwinkler et al. (2014)
*Sebacinacandida*	TUB 020331	USA	KF061278	KF061278	Oberwinkler et al. (2014)
*Sebacinacystidiata*	TUB 020024*	Germany	KF000452	KF000452	Oberwinkler et al. (2014)
*Sebacinacystidiata*	TUB 020025	Germany	KF000453	KF000453	Oberwinkler et al. (2014)
*Sebacinadimitica*	TUB 019987	Austria	KF061271	KF061271	Oberwinkler et al. (2014)
*Sebacinadimitica*	TUB 019988	Austria	KF061272	KF061272	Oberwinkler et al. (2014)
*Sebacinadimitica*	TUB 019989	Austria	KF061273	KF061273	Oberwinkler et al. (2014)
*Sebacinaepigaea*	TUB 019979	Austria	KF000411	KF000411	Oberwinkler et al. (2014)
*Sebacinaepigaea*	TUB 020003	Austria	KF000432	KF000432	Oberwinkler et al. (2014)
*Sebacinaflagelliformis*	TUB 020035	Germany	KF000463	KF000463	Oberwinkler et al. (2014)
*Sebacinaflagelliformis*	TUB 020036*	Germany	KF000464	KF000464	Oberwinkler et al. (2014)
*Sebacinaincrustans*	TUB 019991	Austria	KF000420	KF000420	Oberwinkler et al. (2014)
*Sebacinaincrustans*	TUB 020018	Austria	KF000446	KF000446	Oberwinkler et al. (2014)
*Sebacinaincrustans*	TUB 020030	Germany	KF000458	KF000458	Oberwinkler et al. (2014)
*Sebacinapallida*	TUB 019649	Germany	JQ665564	JQ665564	Oberwinkler et al. (2014)
*Sebacinapallida*	TUB 020209	Germany	KF061276	KF061276	Oberwinkler et al. (2014)
*Serendipitavermifera*	MAFF305835	Australia	DQ983814	DQ983814	Deshmukh et al. (2006)
*Serendipitavermifera*	MAFF305837	Australia	DQ983815	DQ983815	Deshmukh et al. (2006)
*Tremelloscyphadichroa*	Ryvarden 45376	Germany	KF061282	KF061283	Oberwinkler et al. (2014)
*Tremelloscyphadichroa*	VB4210	Germany	KF061280	KF061280	Riess et al. (2014)
*Tremelloscyphagelatinosa*	GG 23605	Germany	AF490394	AF291376	Selosse et al. (2002)
*Tremelloscyphagelatinosa*	VB4212	Germany	JQ012947	–	Bandala et al. (2012)

The sequences newly generated in this study are in bold, and all types are indicated with a superscript asterisk.

The new sequences and reference sequences retrieved from GenBank through BLAST searches (Table 1) were partitioned into ITS1, 5.8S, ITS2, and nLSU. They were aligned with MAFFT v.7.526 (Katoh et al. 2019) and then manually adjusted in BioEdit and Mesquite version 3.04 (Hall 1999; Maddison and Maddison 2017). The separate alignments were then concatenated using PhyloSuite v.1.2.3 (Zhang et al. 2020). Unreliably aligned sections were removed before the analyses, and efforts were made to manually inspect and improve the alignment. We selected *Serendipita* species as the outgroups in the phylogenetic analyses because *Serendipita* species were close to species of Sebacinaceae in the Sebacinales in the study of Oberwinkler et al. (2014). The final alignments and the retrieved topologies were deposited in TreeBASE (http://www.treebase.org) under accessions 32151.

Maximum likelihood (ML) analysis was carried out using the CIPRES Science Gateway by the RaxML-HPC BlackBox tool (Miller et al. 2010). The maximum runtime for the RaxML half-bootstrap analysis was automatically set to 0.25 hours (15 minutes). The remaining parameters were kept at their defaults. Bayesian inference (BI) analysis was performed using MrBayes v3.2.7 (Ronquist et al. 2012) with the best-fit partitioning scheme and substitution models determined in ModelFinder (Kalyaanamoorthy et al. 2017) via the “greedy” algorithm, using branch lengths estimated as “linked” and AICc. Four Markov chains were run for two runs from random starting trees for one million generations until the split deviation frequency value was <0.01, and the trees were sampled at every 1000^th^ generation. The first 25% of the sampled trees were discarded as burn-in, and the remaining ones were used to infer a majority rule consensus and calculate the Bayesian posterior probabilities (BPP) of the clades. Branches were considered significantly supported if they received bootstrap support (BS) for ML and BPPs greater than or equal to 70% (ML) and 0.90 (BPP), respectively.

## ﻿Results

### ﻿Phylogenetic analyses

**Figure 1.**
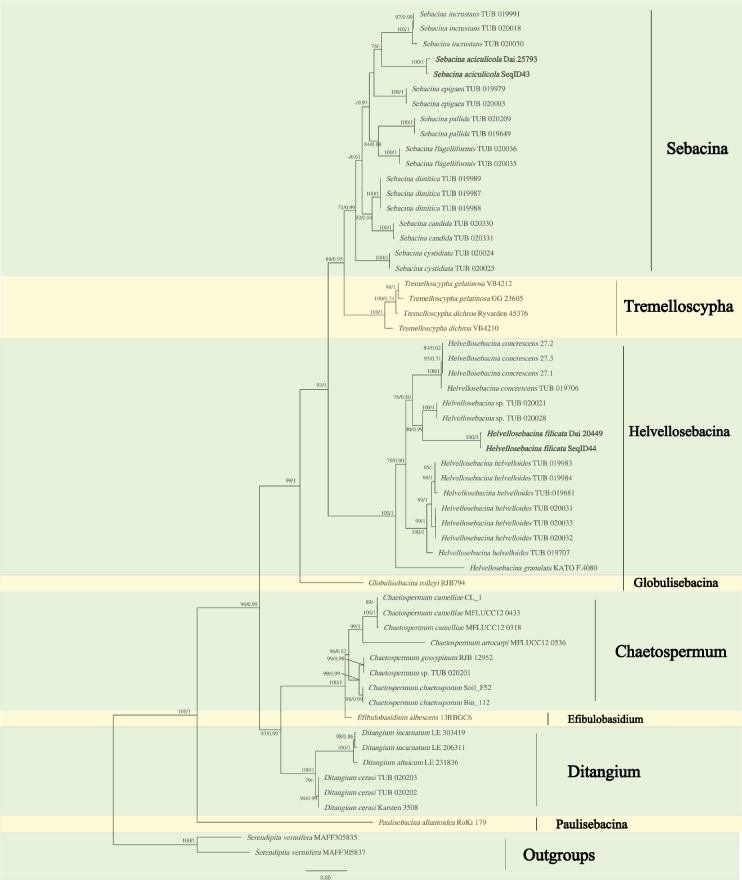
Maximum likelihood (ML) phylogenetic tree illustrating the phylogeny of Sebacinaceae based on the combined ITS+nLSU dataset. Branches are labeled with maximum likelihood bootstrap ≥ 70% and Bayesian posterior probabilities ≥ 0.90, respectively. New species are highlighted in bold.

The combined ITS1-5.8S-ITS2-nLSU dataset included sequences from 57 fungal collections representing 29 taxa of Sebacinaceae, and two samples of *Serendipita* species were used as the outgroups. ModelFinder proposed the models GTR+R3+F for ITS1, SYM+I+G4 for 5.8S, HKY+F+G4 for ITS2, and GTR+R3+F for nLSU for the Bayesian analysis. The BI analysis resulted in an average standard deviation of split frequencies = 0.005400. The ML analysis based on the combined ITS1-5.8S-ITS2-nLSU dataset resulted in a similar topology as the BI analysis, so only the ML tree is presented with the support values from the Bayesian and ML analyses indicated at the nodes (Fig. 1). Our phylogenies based on Bayesian and ML demonstrated that our specimens formed two well-supported monophyletic clades, one in the reasonably well-supported Sebacina clade (71/0.99) and the other in the strongly supported Helvellosebacina clade (100/1). Our sample, Dai20449, together with the culture SeqID44, whose sequence data were downloaded from GenBank, formed a distinct lineage within the *Helvellosebacina* clade (Fig. 1), and another sample, Dai25793, together with the culture SeqID43, whose sequence data were downloaded from GenBank, formed a distinct lineage within the *Sebacina* clade (Fig. 1).

### ﻿Taxonomy

#### ﻿ Helvellosebacina filicata

Taxon classificationFungiSebacinalesSebacinaceae

J.H. Dong, X. Zhang, Y.C. Dai & F. Wu sp. nov.

DBA45381-5561-53EE-B274-24D9DC6E50DE

857594

Figs 2 , 3 , 4

##### Holotype.

China • Yunnan Province, Puer, Puer Forest Park, 17 August 2019, on living ferns, Dai 20449 (holotype, BJFC 032117).

**Figure 2.**
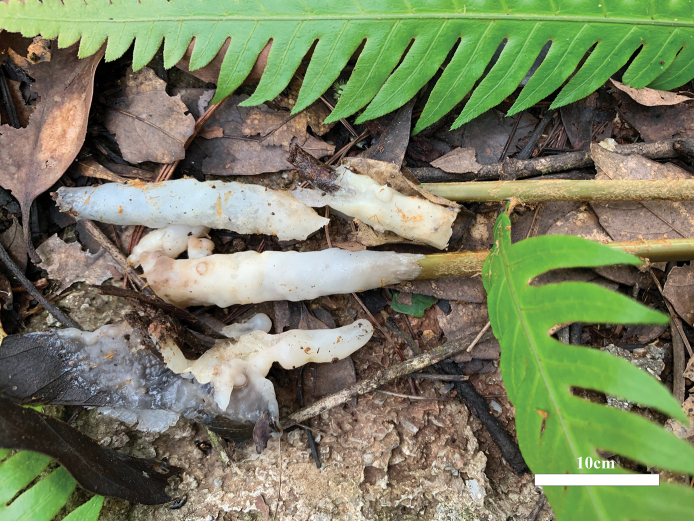
Basidiomata of *Helvellosebacinafilicata* (holotype, Dai 20449).

##### Etymology.

*Filicata* (Lat.): refers to the species growing on living ferns.

**Figure 3.**
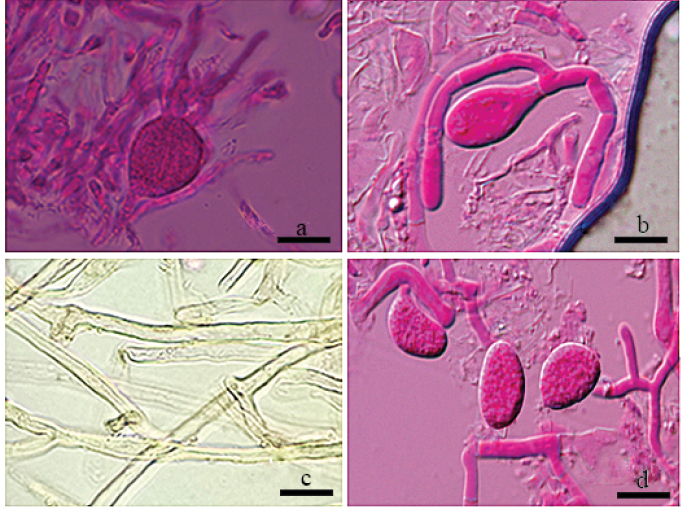
Photos of microscopic structures of *Helvellosebacinafilicata* (holotype, Dai 20449) **a, b** basidia **c** hyphae **d** basidiospores. Scale bars: 10 μm (**a–c**); 5 μm (**d**).

##### Description.

***Basidiomata*.** Annual, resupinate, amorphous, closely adnate on substrate, widely effused, easily separable, cartilaginous to gelatinous, white to cream, sometimes semi-transparent when fresh, up to 12 cm diam and 0.05 cm thick, distinctly shrinking to a film and becoming fawn to clay-buff when dry.

**Figure 4.**
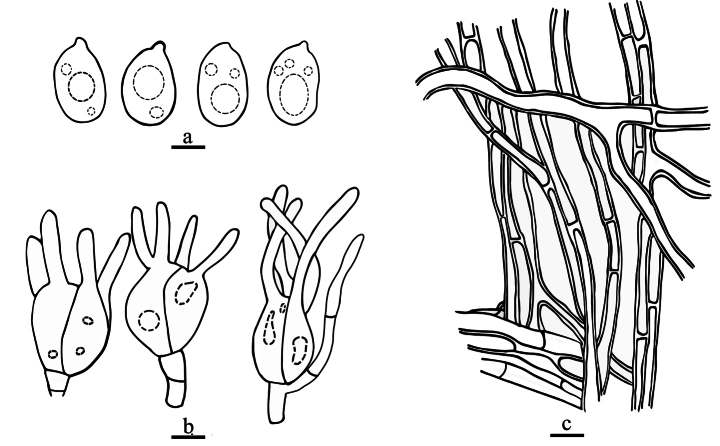
Drawings of microscopic structures of *Helvellosebacinafilicata* (holotype, Dai 20449) **a** basidiospores **b** basidia **c** hyphae. Scale bars: 5 μm (**a, c**); 10 μm (**b**).

***Hyphal structure*.** Hyphal system monomitic; hyphae simple or branched, sometimes flexuous, hyaline, thin-walled or thick-walled, without clamp connections, 2.6–3.8 µm diam., embedded in hymenium and subhymenium.

***Hymenium*.** Cystidia absent; hyphidia (dikaryophyses) present, unbranched, with several simple septa, usually derived from the same hyphae with probasidia; probasidia pyriform to subglobose; mature basidia thin-walled, obovate to subglobose, 12–16.8 × 9.8–13.7 μm, longitudinally septate, 4-celled, usually with oil drops; sterigmata up to 52.7 μm long, 1.4–2.7 μm diam., with tapered apex.

***Basidiospores*.** Hyaline, thin-walled, smooth, broadly ellipsoid to ovoid, apiculate, usually with several large oil drops, (7.9–)8.6–9.9(–11.1) × (5.1–)5.8–7.6(–8.3) μm, L = 9.2 μm, W = 6.8 μm, Q = 1.35 (n = 40/1).

#### ﻿ Sebacina aciculicola

Taxon classificationFungiSebacinalesSebacinaceae

J.H. Dong, X. Zhang, Y.C. Dai & F. Wu sp. nov.

94663D0A-665A-59D8-9E4C-E26BAB46B742

857598

Figs 5 , 6 , 7

##### Holotype.

China • Guizhou Province, Guiyang, Qianlingshan Park, 21 August 2023, bark of *Pinusmassoniana*, Dai 25793 (holotype, BJFC 043342).

**Figure 5.**
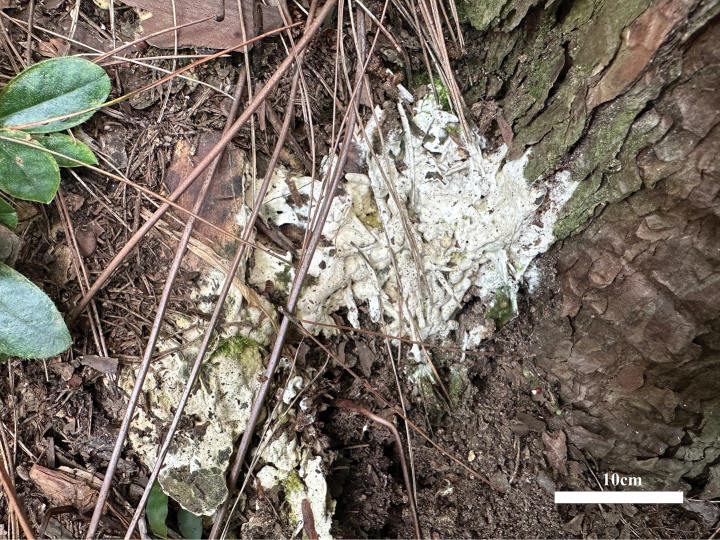
Basidiomata of *Sebacinaaciculicola* (holotype, Dai 25793).

##### Etymology.

*Aciculicola* (Lat.): refers to the species growing on needles and bark of *Pinusmassoniana*.

**Figure 6.**
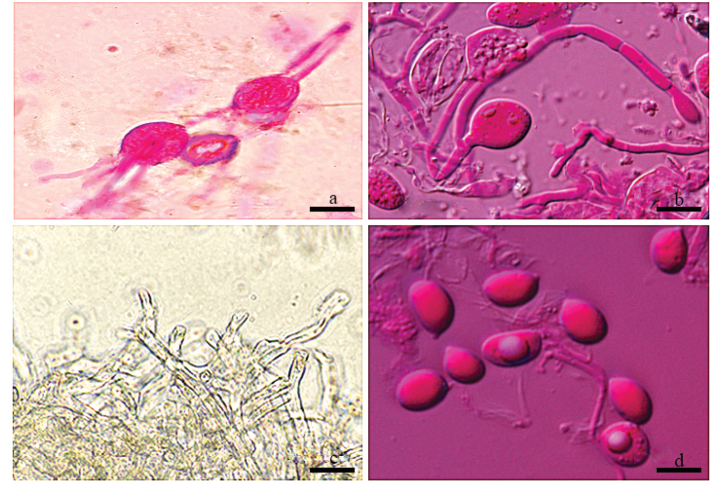
Photos of microscopic structures of *Sebacinaaciculicola* (holotype, Dai 25793) **a, b** basidia **c** hyphae **d** basidiospores. Scale bars: 10 μm (**a–c**); 5 μm (**d**).

##### Description.

***Basidiomata*.** Annual, resupinate, closely adnate on substrate, widely effused, not easily separable, coriaceous, white to cream when fresh, up to 15 cm diam and 0.1 cm thick, color and structure almost unchanged when dry; margin gradually thinning out, concolorous with the center.

**Figure 7.**
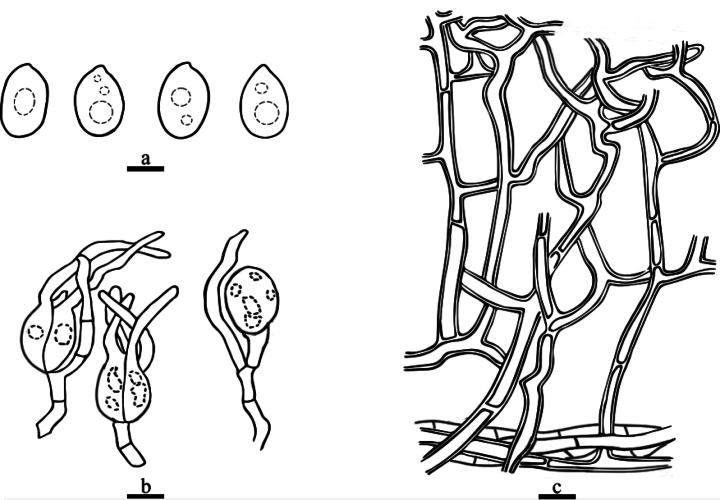
Drawings of microscopic structures of *Sebacinaaciculicola* (holotype, Dai 25793) **a** basidiospores **b** basidia **c** hyphae. Scale bars: 5 μm (**a, c**); 10 μm (**b**).

***Hyphal structure*.** Hyphal system monomitic; hyphae simple or branched, sometimes flexuous, hyaline, thin-walled or thick-walled, smooth, without clamp connections, 2.0–5.0 µm diam., embedded in hymenium and subhymenium.

***Hymenium*.** Cystidia absent; hyphidia (dikaryophyses) present, unbranched, with several simple septa, usually derived from the same hyphae with probasidia; probasidia pyriform to subglobose; mature basidia thin-walled, obovate to subglobose, stalked, 16–22.8 × 11–14 µm, longitudinally septate, 4-celled, usually with oil drops; sterigmata up to 62.0 μm long, 2.0–3.5 μm diam., with tapered apex.

***Basidiospores*.** Hyaline, thin-walled, smooth, broadly ellipsoid to ovoid, apiculate, usually with several large oil drops, (9.3–)10.2–12.9(–13.7) × (6.2–)6.3–8.7(–9.1) µm, L = 10.9 µm, W = 7.4 µm, Q = 1.47 (n = 40/1), germinating by germination tubes.

## ﻿Discussion

*Helvellosebacinafilicata* and *Sebacinaaciculicola* are described as two new species of the Sebacinaceae using molecular and morphological data. *H.filicata* was discovered in the subtropical area of Yunnan Province in China, and it is the fourth species of this genus. In our phylogenies, *H.filicata* formed an independent, well-supported lineage most closely related to an unnamed *Helvellosebacina* sp. recovered by Oberwinkler et al. (2014) (Fig. 1). The genetic distance between *H.filicata* and *Helvellosebacina* sp. in their ITS sequences is above 7%. However, detailed morphological characteristics of *Helvellosebacina* sp. were not provided by Oberwinkler et al. (2014). Therefore, we are unable to provide a direct morphological comparison between *H.filicata* and *Helvellosebacina* sp. at this time. In addition, *H.concrescens* and *H.granulata* E. Sesli differ from *H.filicata* by their irregular, granular, whitish-to-beige with a very light pink tint hymenial surface when fresh (Roberts 2003; Sesli 2021). *H.helvelloides* (Schwein.) Oberw. et al. differs from *H.filicata* by spongy, effused, convex, white or buff merulioid-helvelloid basidiomata, distinctly larger basidia (20–25 × 15 μm vs. 12–16.8 × 9.8–13.7 μm), and basidiospores (12–13 × 6 μm vs. 8.6–9.9 × 5.8–7.6 μm) (Oberwinkler et al. 2014; Sesli 2021).

*Sebacinaaciculicola* was discovered in the subtropical area of Guizhou Province of China. Morphologically, *S.aciculicola* is similar to *S.epigaea* (Berk. & Broome) Bourdot & Galzin and *S.incrustans* by sharing resupinate, encrusting substrates basidiomata when fresh, but *S.epigaea* differs from *S.aciculicola* by its gelatinous basidiomata, branched dikaryophyses, and basidiospore germination with star-like secondary spores. Our new species, *S.aciculicola*, formed an independent, well-supported lineage sister to *S.incrustans* (Fig. 1). The ITS sequences are approximately 7% different between the two taxa. While the basidiomata of *S.incrustans* are described as dry to waxy and may vary from cream to greyish when fresh, such color traits may overlap with other congeners under different environmental conditions. *S.incrustans* furthermore has branched dikaryophyses (Oberwinkler et al. 2014). *S.aciculicola* can also be distinguished from *S.incrustans* by its distinctly larger basidia (16–22.8 × 11–14 μm vs. 15–18 × 10–12.5 μm) and by its slightly larger basidiospores (10.2–12.9 × 6.3–8.7 μm vs. 10–12.5 × 6–8.5 μm).

The family Sebacinaceae forms fruiting bodies under different environmental conditions and associates with diverse plant hosts (Wei and Agerer 2011), and current studies have shown that this family is one of the most species-rich and abundant nonspecific ectomycorrhizal (ECM) fungal lineages from temperate and tropical regions (Weiß et al. 2016). The basidiomata and host preferences of our two new species show similarities to other wood-decaying fungi documented in China (Dai 2010, 2011, 2012; Wang et al. 2023, 2024; Zhao et al. 2024), and they have been found before in the form of two GenBank sequences (SeqID43 and SeqID44). The ecological roles of the two new species remain uncertain due to the limited number of specimens and their collection from fern and pine bark, and the two extant GenBank records did not offer any further information. Based on the substrate and host associations, these species are likely to be saprophytic. Since Sebacinaceae comprises both ECM and saprophytic fungi, further studies will be needed to establish the ecological functions of the two new species. Despite the ecological and evolutionary significance of Sebacinaceae, Chinese species in this family are still not well known, especially in subtropical and tropical regions. The two new species described here add depth to our of understanding of Sebacinaceae in China and beyond.

## Supplementary Material

XML Treatment for Helvellosebacina filicata

XML Treatment for Sebacina aciculicola

## ﻿Additional information

### Author ORCIDs

Jia-Hui Dong https://orcid.org/0009-0005-8456-3699

Xin Zhang https://orcid.org/0009-0005-8363-7852

Qian-Xin Guan https://orcid.org/0000-0002-7072-080X

Fang Wu https://orcid.org/0000-0002-1455-6486

### Data availability

All of the data that support the findings of this study are available in the main text.
